# Invasive African fig flies ( *Zaprionus indianus* ) have reduced cold acclimation ability compared to cosmopolitan drosophilid species

**DOI:** 10.17912/micropub.biology.002245

**Published:** 2026-06-17

**Authors:** Mia Hollekim, Lucy Smith, Priscilla Erickson

**Affiliations:** 1 Biology, University of Richmond, Richmond, VA, US

## Abstract

*Zaprionus indianus* , an invasive species of fruit fly originating from Africa, has successfully inhabited eastern North America but is thought to be seasonally limited by cold temperatures. We compared the cold acclimation abilities of *Z. indianus* to six drosophilid species. Each species’ survival at -5°C was tested following acclimation periods at 10°C, 15°C, and 25°C. We found that *Z. indianus* had a similar baseline cold tolerance to other cosmopolitan species and acclimated following treatment at 15°C. Overall, cold acclimation in *Z. indianus* was better than tropical species but weaker than some cosmopolitan species, suggesting cold may restrict *Z. indianus* populations.

**Figure 1. Seven species of drosophilids vary in baseline cold tolerance and cold acclimation:** *Z. indianus* performance was compared against six drosophilid species to compare cold acclimation capabilities. Each species was pre-exposed to cool temperatures (15°C and 10°C) or a control treatment (25°C) for four days, followed by a -5°C freeze exposure for varying times (0-900 minutes, depending on the species). Survival curves (A) were used to calculate LT₅₀ (B) which is defined as the time of cold exposure after which 50% of individuals remained alive. Survival curves and LT₅₀ graphs are displayed with SEM error bars. Significant differences within each species between pre-treatment groups are indicated at the top with black asterisks (P < 0.05 after Bonferoni correction). Significant differences between each species and *Z. indianus* receiving the same temperature treatment are marked with colored asterisks (P < 0.05 after Bonferoni correction). Sample size varies across species and treatments (average n=46 flies per data point, range = 9-90). Note that *D. virilis* has longer exposure times to -5°C in comparison to the other six species.

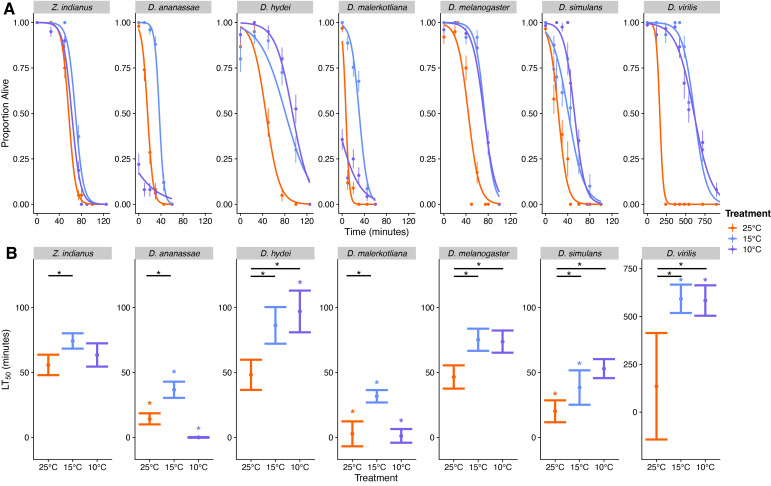



## Description

Invasive species often efficiently acclimatize to new environments but are still susceptible to biotic and abiotic limitations (Davidson et al., 2011). Temperature can limit invasive ectotherms’ ability to survive and spread by affecting reproduction, metabolic rates, growth, and survival (MacMillan et al., 2015; Ørsted et al., 2024; Terblanche et al., 2007). For example, upon exposure to cold, non-freezing temperatures, many insects enter paralyzed chill comas affecting muscles and neurons (Davis et al., 2021). Thus, exposure to lower temperatures can be limiting for invasive species from tropical or subtropical areas, where cold tolerance is often weak (Turnock & Fields, 2005). Cold tolerance is determined by two primary characteristics: baseline cold tolerance is the inherent ability to survive cold stress without acclimatization, while cold acclimation is a phenotypically plastic response that occurs when organisms are preexposed to non-lethal cold stress (Hine, 2019). Within a species, as studied in *Drosophila melanogaster,* both features of cold tolerance vary due to genetics (Czajka & Lee, 1990; Hine, 2019; MacMillan et al., 2016; Pool et al., 2017). *D. melanogaster* has successfully adapted to temperate environments over several thousand years due to changes in both baseline cold tolerance and acclimation abilities (MacMillan et al., 2016; Nyamukondiwa et al., 2011; Sprengelmeyer et al., 2020). However, some invasive drosophilids have been introduced to temperate environments much more recently, raising the question of whether they have the physiological capacity to survive temperate winters.

*Zaprionus indianus* , the African Fig fly, originated in central Africa and has recently dispersed to the Americas. First detected in Brazil in 1999 and Florida in 2005, it has spread throughout the east coast of North America and thrives in commercial agriculture settings (Commar et al., 2012; Gray et al., 2025; Linde et al., 2006; Pfeiffer et al., 2019; Yassin et al., 2008). *Z. indianus* is an economic pest, as eggs are laid in both rotten and healthy fruit, damaging crops (EFSA Panel on Plant Health (PLH) et al., 2022; Linde et al., 2006). This species has rapidly adapted to North American environments, with evidence of genetic and phenotypic changes (Erickson et al., 2025; Gray, Rakes, et al., 2025; Pfeiffer et al., 2019). *Z. indianus* has limited cold tolerance, with low survival after exposure to 10-12°C for 20 days (Lavagnino et al., 2020). Field data suggest that *Z. indianus* likely does not overwinter in temperate environments (Gray, Stephenson, et al., 2025; Kohlmeier & Kohlmeier, 2025; Rakes et al., 2023), but it could become permanently established in the cold temperatures of eastern North America through a combination of phenotypic plasticity and its potential for rapid evolution. To address the threat of future permanent establishment, the cold acclimation abilities of *Z. indianus* were compared to four cosmopolitan ( *D. melanogaster, D. simulans, D. virilis,* and *D. hydei)* and two tropical ( *D. ananassae* and *D. malerkotliana* ) drosophilid species in the context of their size, ecological, and geographical differences.

The baseline cold tolerance of *Z. indianus* to a -5°C cold shock following a control treatment at room temperature (25°C) was higher than that of *D. malerkotliana* , *D. simulans* , and *D. ananassae* (Bonferoni-corrected P < 0.05), and did not differ from the other three species (Bonferoni-corrected P > 0.05) (Figure 1). In most species, flies exposed to 10°C or 15°C cold acclimation treatments had improved survival following cold shock ( Figure 1A ). However, when comparing LT _50_ values (the time of cold exposure that killed 50% of flies), *Z. indianus* had less cold acclimation capabilities compared to some cosmopolitan drosopholid species ( Figure 1B ). For example, *Z. indianus* exhibited a 1.3-fold increase of baseline LT _50_ following treatment at 15°C, while *D. virilis* exhibited a 4.4-fold increase. The reduced ability to acclimate to cold temperatures may lead to reduced survival in winter compared to some cosmopolitan species.

Surprisingly, the species that exhibited the most similar cold acclimation capabilities to *Z. indianus* was *D. melanogaster.* This result is unexpected because *D. melanogaster* overwinters in temperate environments while *Z. indianus* is not thought to (Ives, 1970; Pfeiffer et al., 2019; Kohlmeier & Kohlmeier, 2025). We expected to see more similarities between *Z. indianus and D. simulans* as they are both thought to perish during winter months (Machado et al., 2016; Pfeiffer et al., 2019). There were no significant differences in LT _50_ between *Z. indianus* and *D. melanogaster* receiving the same temperature treatments ( Figure 1B ), and *Z. indianus* had a significantly higher LT _50_ than *D. simulans* for the 25°C and 15°C treatments. Genetic analysis between the summer and winter months suggest an overwintering capability within *D. melanogaster* (Ives, 1970), likely via cold acclimation that occurs in the field (Stone et al., 2020) and rewires the transcriptome (MacMillan et al., 2016). The similarity in LT _50_ between *Z. indianus* and *D. melanogaster* indicates the capability of *Z. indianus* for cold acclimation is higher than predicted given its apparent lack of overwintering. However, other physiological capacities beyond cold acclimation, such as diapause, may contribute to *D. melanogaster* ’s apparently better overwintering survival (Schmidt & Conde, 2006), though *Z. indianus* is also known to diapause (Lavagnino et al., 2020).

We also observed a lower tolerance for 10°C treatments in *Z. indianus* and two tropical species, *D. ananassae* and *D. malerkotliana.* Previous literature demonstrates that *D. ananassae* cannot be reared below 16°C (Morin et al., 1997) and *D. malerkotliana* can acclimatize to the mildly cold temperature of 17°C (Parkash et al., 2021). These observed patterns relate to their origin in tropical environments (Werner et al., 2018). The LT _50_ values for all three species peaked at 15°C, but 10°C treatments were approximately as lethal as the -5°C shock treatment following no acclimation. Many of the *D. ananassa* e and *D. malerkotliana* held at 10°C were deceased prior to the freeze challenge, suggesting that four days at 10°C was detrimental. Because the pre-treatment likely killed the least cold tolerant *D. ananassae* and *D. malerkotliana* , we may have overestimated freeze survival of this selected subset; *Z. indianus* may be even more cold tolerant relative to these tropical species. The decrease in freeze survival of *Z. indianus* following 10°C treatment was less extreme than that observed in the tropical species and was not driven by mortality during the acclimation period. Therefore, in *Z. indianus,* exposure to temperatures below 15°C may exert negative effects rather than promoting acclimation.

In contrast to the tropical species and our findings for *Z. indianus, D. virilis* demonstrated the greatest cold-hardening capability across all species, with an over 4-fold increase in LT _50_ when cold acclimated. Although *D. hydei* also demonstrated significant cold acclimation (~2-fold increase in LT _50_ when cold acclimated), the *D. virilis* individuals were more cold tolerant in terms of the total time of exposure to -5°C they survived. This result indicates that *D. virilis* has a higher capability of surviving in colder environments. This pattern is consistent with their ability to enter a prolonged chill coma, allowing them to withstand cold temperatures for extended periods (Hoikkala & Poikela, 2022; Vesala et al., 2012; Yusuf et al., 2022).

One limitation of this study is that the *Z. indianus* were collected from a mid-latitude location during the summer and reared in the lab for several years. Given the high genetic variation of *Z. indianus* and its adaptive potential (Erickson et al., 2025; Gray, Rakes et al. 2025), we may have obtained different results had we used flies collected at another time or location. We hypothesize that flies captured in Virginia are the descendants of flies that successfully overwintered in the mild climates of northern Florida or southern Georgia and were introduced northwards. Flies collected from southern Florida might demonstrate less cold tolerance. Another limitation is the varied sources of the species we compared. Some were collected from the wild recently, whereas others have been in laboratory culture. In general, insects reared in the lab lose their stress tolerance due to relaxed selection in stable laboratory environments (Hoffmann & Ross, 2018). Therefore, we may have underestimated the cold tolerance of all species, but especially for *D. melanogaster* and *D. virilis* that were long-held lab strains.

In conclusion, *Z. indianus* demonstrated a baseline cold tolerance similar to other cosmopolitan species and the highest similarity in cold acclimation ability to *D. melanogaster.* Overall, the *Z. indianus* performed better than expected given its limited overwintering, however increased mortality following the 10°C treatment and freezing suggests that prolonged cold exposure is physiologically taxing for *Z. indianus* . The performance of *Z. indianus* did not differ from *D. melanogaster* at any temperature, but we see a similar pattern of reduced survival at 10°C relative to 15°C in *Z. indianus, D. malerkotliana,* and *D. ananassae.* The negative effect of lower temperatures may explain why *D. melanogaster* can overwinter and is found readily in the early spring, whereas *Z. indianus* are not observed in the winter months or early spring and are thought to be killed by cold temperatures. Additional work exploring the thermal limits and cold acclimation abilities of *Z. indianus* across a broader range of thermal treatments will help to better predict the establishment and persistence of this crop pest in temperate environments.

## Methods

Lab-reared *Z. indianus* derived from a wild population collected in Charlottesville, Virginia in 2019 were compared against six other drosophilid species. *D. simulans* were collected in Florida in 2020. *D. ananassae* and *D. malerkotliana* were collected from Florida in 2024. *D. hydei* was collected from Virginia in 2023. *D. melanogaster* was the Canton-S lab strain obtained from the Bloomington Stock Center. *D. virilis* was strain 15010‑1051.87 obtained from the Drosophila Species Stock Center. All experiments were conducted in summer 2025.

Species were chosen based on climate, body size, and habitat to compare to *Z. indianus* . *D. melanogaster* , *D. hydei* , *D. simulans* and *D. virilis* are all considered widespread cosmopolitan species. *D. melanogaster,* a subtropical African species, is established nearly worldwide and can withstand cold temperatures due to physiological changes from extreme cold tolerance and rapid cold acclimation (MacMillan et al., 2016). Conversely *, D. simulans,* also from East Africa, has less genetic and physiological flexibility to cold environmental stressors in temperate areas (Werner et al., 2018). *D. virilis,* a larger species from East Asia (Throckmorton, 1982) can withstand decreasing temperatures regulated by gene expression and metabolic responses (MacMillan et al., 2016; Vesala et al., 2012). *D. hydei,* from South America, has moderate cold tolerance due to its origins and consistent with its distribution (Alruiz et al., 2022). In contrast to these cosmopolitan species, *D. ananassae* and *D. malerkotliana* are restricted to tropical and subtropical locales. *D. ananassae,* a South Asian species, has some physiological ability to acclimate to cold but has reduced tolerance in comparison to the other species (Yılmaz et al., 2025). Lastly, *D. malerkotliana,* another tropical South Asian species, inhabits warm areas and is cold-sensitive but is cold-tolerant with changing seasons (Parkash et al., 2014). Overall, these species from tropical to temperate ecologies were selected to understand cold-tolerance and acclimation ability in relation to historical origins.

All flies were reared in an incubator at 25°C with 50% humidity in a 14L:10D light cycle. Adult flies were placed in egg-laying chambers containing 3% agar in grape juice and a paste made of active yeast and water to induce mating. After a 24-hour incubation period, adult flies were removed, and the eggs were allowed a 24-72 hour (depending on the species) period to hatch before collection. The hatched larvae were collected and placed into food vials containing 10 mL cornmeal-molasses medium. Larger drosophilid species, including *D. virilis, D. hydei,* and *Z. indianus,* were reared at a density of 30 larvae per food container, while the smaller species, *D. ananassae, D. malerkotliana, D. melanogaster,* and *D. simulans,* were reared with 50 larvae per food container. Larvae developed at 25°C and 50% relative humidity until their emergence as adults. Emerged flies were sorted for treatment into vials of 10 females. Vials of flies were randomly assigned cold acclimation treatment temperatures of 15°C and 10°C, and a randomly assigned control group was reared at 25°C to determine baseline cold tolerance. All flies were acclimated for four days.

A tank containing 7-8 cm saturated salt water (~360 g/L NaCl to prevent freezing) was placed into a chest freezer with air temperature maintained at -5°C using a brewing thermostat. Weights were used to submerge test tube racks in the water. After the four-day incubation period, flies were transferred into 5 mL polystyrene culture tubes with a piece of cotton below the lid to prevent the flies from getting stuck or crushed. Any flies that were dead following the cold acclimation treatment were recorded prior to shock treatment, but were considered dead for the purpose of data analysis. The tubes were then placed into the saltwater bath treatment. Tubes were removed at various time points for each species based on preliminary assays. *Z. indianus* and *D. hydei* were assessed from 0 to 125 minutes with 25-minute intervals. *D. ananassee* and *D. malerkotliana* were measured from 0 to 60 minutes with 10- and 15-minute intervals, respectively. *D. melanogaster* was assessed from 0 to 100 minutes using 20-minute intervals. *D. simulans* spanned from 0 to 75 minutes with 15-minute intervals. Finally, *D. virilis* was tested from 360 to 1,080 minutes, with flies extracted every 180 minutes (with a negative control included at 0 minutes). All species were tested with at least four replicate survival curves. On some occasions, we changed the time points slightly between experimental replicates to better capture variation in survival over time, resulting in variable sample sizes per time point. Flies were given a 24 hour recovery period, and the number of flies dead/alive was recorded. Dead flies were defined as individuals who could not stand and walk on their own after agitation. An average of 1,048 flies of each species were tested (range = 691-1,240).

The *drm* and *ED* functions of the *drc* package (v. 3.0-1) (Ritz et al., 2015) were used in R v 4.5.0 (R Core Team, 2025) to calculate the LT _50_ : the time at in which 50% of the flies had died due to the cold treatment based on a binomial generalized linear model with survival as the response variable and species, treatment, and exposure time to -5°C as predictors. We combined all replicate vials for each time point for each treatment and species into a single survival curve; models that kept data separate by vial failed to converge due to the small sample size of 10 flies per vial and the large number of 0% and 100% survival vials. We acknowledge that combining all data without correcting for individual vials as a random effect results in pseudoreplication in the dataset. The *compParm* function was used to calculate P-values for comparisons of treatment groups and species. Bonferoni corrections were used to determine significant differences for comparisons between treatments within species (N=21 tests, α = 0.05/21 = 0.0024) and comparisons of *Z. indianus* to other species receiving the same treatment (N=18 tests, α = 0.05/18 = 0.0028). *ggplot2* was used to generate graphics (Wickham, 2016).
